# Effects of script similarity on bilingual advantages in executive control are likely to be negligible or null

**DOI:** 10.3389/fpsyg.2014.01539

**Published:** 2015-01-07

**Authors:** Kenneth R. Paap, Jack Darrow, Chirag Dalibar, Hunter A. Johnson

**Affiliations:** ^1^Language Attention and Cognitive Engineering Lab, Department of Psychology, San Francisco State UniversitySan Francisco, CA, USA; ^2^Department of Philosophy, San Francisco State UniversitySan Francisco, CA, USA

**Keywords:** bilingual advantages, executive functioning, cognition, orthography, script

Coderre and van Heuven (2014) concluded that the orthographic overlap between a bilingual's two languages is an important variable to consider in investigations of bilingual executive control (EC) abilities. Since greater overlap creates more cross-linguistic activation and (presumably) increases the daily demands of cognitive control Coderre and van Heuven hypothesize that similar-script bilinguals will have more effective domain-general EC. In their study German-English (GE) bilinguals were identified as a having high similarity, Polish-English (PE) as having moderate similarity, and Arabic-English (AE) as having low similarity; there was also a group of monolingual English speakers. All groups participated in both a Stroop and Simon task. The bilinguals did one Stroop session with printed words from L1 and a second session with L2. The interference effect (incongruent RT − neutral RT) was taken as a measure of general inhibitory control whereas global RT (mean RT across all trial types) was taken as a measure of “monitoring for conflict.”

The hypothesis that orthographic overlap enhances EC efficiency (marked by smaller interference effects or faster global RTs) predicts this ordering: GE < PE < AE. Each measure (global RT, interference) affords three pairwise comparisons (viz., GE<PE, GE<AE, PE<AE) for each of the three tasks (Simon, L1 Stroop, and L2 Stroop) for a total of 18 pairwise tests. Using global RT as a measure of EC, the predictions are supported by only three of the nine comparisons. Even worse, the predictions for the interference effects were not supported by any of the nine comparisons: eight of the comparisons were null and the significant advantage of AEs over PEs in the L1 Stroop task contradicts the prediction. In total 3 of the 18 comparisons support the hypothesis that script similarity enhances EC. The assumption that the three significant differences in global RT that do occur between the bilingual groups should be interpreted as differences in EC is further eroded because none of the bilingual groups showed significantly faster global RTs compared to the monolinguals. With three different tasks and three different groups of bilinguals there were nine such opportunities. In summary, the entire case that orthographic overlap enhances EC is built on the three comparisons out of nine where lower similarity bilinguals were significantly slower in global RT compared to bilinguals with greater script similarity. But global RT is a very impure measure of EC (Paap and Greenberg, 2013) and, as discussed by Coderre and van Heuven, there could be many reasons why the AE bilinguals were slower than the other groups^1^. This spotty evidence for a script similarity effect coupled with the absence of any bilingual advantages (in comparison to the monolinguals) seriously challenges the plausibility of the conclusion that orthographic overlap has modulated EC. Focussing on a small number of comparisons that “work” while letting many inconvenient results recede into the background is a common characteristic of confirmation bias (Paap, 2014; Paap and Liu, 2014).

Given the weakness of the evidence presented by Coderre and van Heuven for the hypothesis that orthographic overlap enhances EC it is not surprising that others report a combination of null and opposing results. Coderre and van Heuven acknowledge that their finding of longer global RTs for different-script bilinguals contradicts the findings of Bialystok et al. (2005) and Linck et al. (2005). Bialystok et al. found that different-script bilinguals (Cantonese-English) had faster global RTs compared to French-English bilinguals. Linck et al. (2005) reported that different-script (Japanese-English) bilinguals had smaller Simon interference effects compared to Spanish-English bilinguals. Without providing any theoretical rationale for the opposing results Coderre and van Heuven speculate that “This could indicate that writing system, rather than script, is the more influential factor…” (Coderre and van Heuven, 2014, p. 11).

This possibility was put to a test using the composite database described in Paap et al. (2014). A simple group analysis partitioned 160 English-other bilinguals into three groups: (1) a “same-alphabet” group (whose other language is also written in the Roman alphabet, *n* = 107), (2) a “different-alphabet/script” group (whose other language is either written in a different abecedary, an abjad, or an abugida; *n* = 25), and (3) a logographic group (*n* = 28). There was also a group of English speaking monolinguals (*n* = 114) with very little or no exposure to another language. Most of the participants in the composite database completed a Simon task, flanker task, and a color-shape switching task. The tasks are described in detail in both Paap and Greenberg (2013) and Paap et al. (2014).

Of primary interest are the interference scores and global RT scores in the Simon task. A One Way ANOVA on the global RT scores for each of the four groups was not significant, *F*_(3, 270)_ = 1.32, *p* = 0.269. The corresponding ANOVA on the Simon interference scores was significant, *F*_(3, 270)_ = 2.78, *p* = 0.042. Exploring the significant main effect of group with Bonferroni comparisons showed that the 26 ms Simon effect for the monolingual group was significantly less than the 38 ms effect for the same-alphabet group, *p* = 0.026. Thus, the bilinguals in the same-alphabet group were at a *disadvantage* compared to the monolinguals. More relevant for present purposes, there were no differences across the bilingual groups and, consequently no evidence that could reconcile the important differences between Coderre and van Heuven (script *similarity* enhances global RT), Bialystok et al. (script *dissimilarity* enhances global RT), and Linck et al. (script *dissimilarity* enhances inhibitory control). The group means and 95% confidence intervals for each analysis are shown in Figure 1.

**Figure 1:**
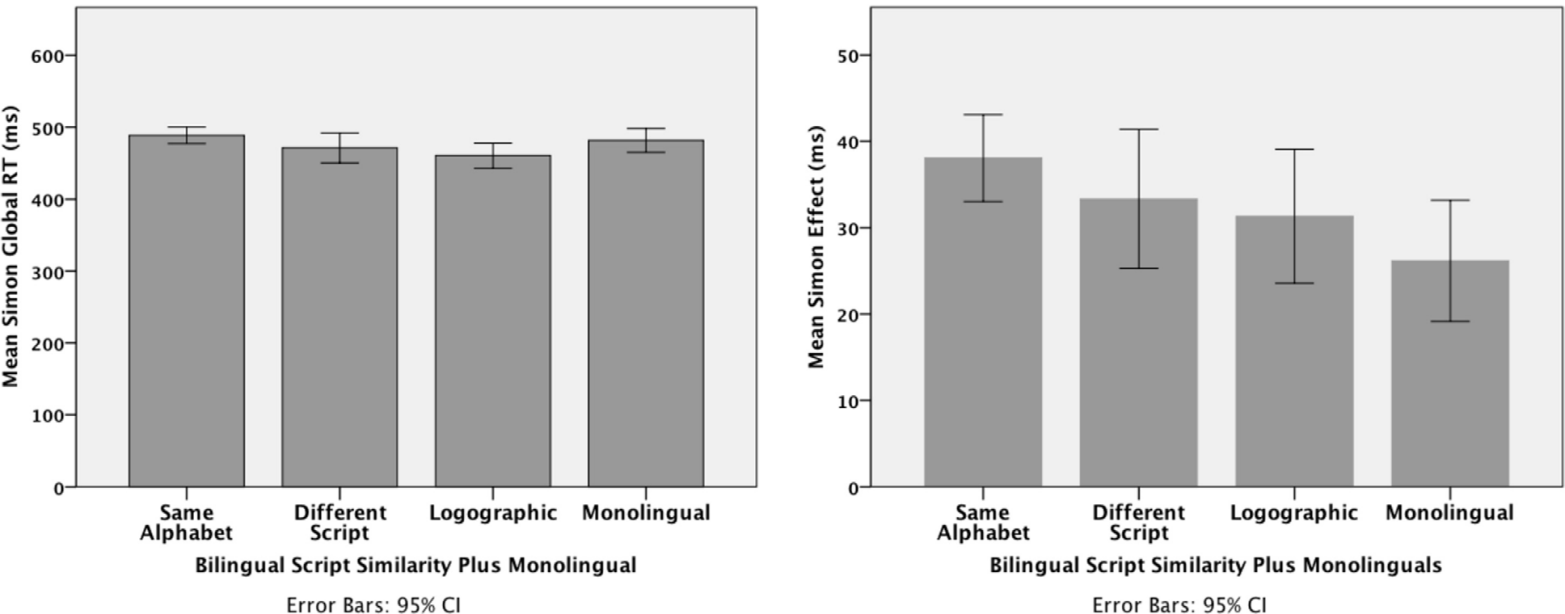
**The effect of script similarity on global RT (left panel) and interference scores (right panel) for the Simon-task data from Paap et al. (2014)**.

One challenge in investigating the role of script similarity is the lack of an objective and quantitative measure of similarity. We made an exploratory first attempt by focusing only on the 119 English-other bilinguals whose languages used alphabets. Orthographic overlap was operationally defined as follows. One point was awarded for each lowercase and uppercase English letter that was shared with the other language for a maximum possible score of 52. One point was subtracted for each “extra” lowercase letter in the other language, e.g., the tilde ñ reduced the overlap score for Tagalog to 51. The overlap scores ranged from 18 (Greek) to 52 (Pangasinan, Indonesian, Malay) with a median of 88 (Spanish). The correlation between this measure of orthographic overlap and the Simon interference effect was not significant, *r*_(167)_ = + 0.111, *p* = 0.153, but the correlation with Simon global RT was, *r*_(167)_ = + 0.118, *p* = 0.015. However, contrary to the prediction based on competition between the lexicons the positive correlation indicates that global RT increases as orthographic overlap increases. It would be risky to interpret the positive correlation with global RT as reflecting a relationship with the monitoring component of EF because global RT can be contaminated by differences in the speed of perceptual and motor processing. An arguably better measure of monitoring (Paap and Greenberg, 2013) is the difference between the mean RT on the congruent trials of a standard mixed block and a baseline block where left-right spatial conflict never occurs. For this measure the correlation is near zero, *r*_(129)_ = −0.023.

In conclusion, investigations of script similarity show a very inconsistent pattern of results across various measures of EC with null results dominating a far smaller number of outcomes that in one case favored bilinguals reading similar scripts (Coderre and van Heuven), but in other cases favored bilinguals reading dissimilar scripts (Bialystok et al., 2005; Linck et al., 2005).

Given that the evidence for an effect of script similarity on EC is both weak and inconsistent it may be that it simply has no effect on domain-general EC. On the other hand, if there is a real effect of script similarity the challenges to revealing it are even greater in comparison to other types of specific bilingual experience such as age-of-acquisition of L2, L2/L1 proficiency, and frequency of switching. One problem is the absence of a quantitative and objective measure of script similarity. To address this problem we have offered a metric of orthographic overlap that credits matching letters and debits mismatching letters, but this approach does not readily extend to abjad, abugida, logographic, and mixed scripts. Establishing consistent results of script similarity is likely to be further handicapped by the difficulty in matching groups for cultural differences ranging from parenting practices to values associated with individualism vs. collectivism (Carlson and Meltzoff, 2008; Morton and Carlson, 2014; Paap, 2015) as these cultural differences are likely to systematically vary across the types of writing systems.

As revealed in this discussion of script-similarity and also by the factors examined by Paap et al. (2014), the efforts of the current Zeitgeist to discover the critical conditions for consistently observing bilingual advantages in EC ironically appear to be generating even more conflict and less coherence in the published database.

## Conflict of interest statement

The authors declare that the research was conducted in the absence of any commercial or financial relationships that could be construed as a potential conflict of interest.

## Footnotes

^1^These confounds are more likely to occur when a study uses risky small n's (Paap and Sawi, 2014). In the Coderre and van Heuven study the language groups ranged from 17 to 22 participants.
